# Conservation of both hematocrit and liver regeneration in hepatectomies: a vascular occlusion approach in rats

**DOI:** 10.1590/0102-672020190001e1

**Published:** 2020-03-30

**Authors:** Eduardo Augustus MALINOWSKI, Jorge Eduardo Fouto MATIAS, Ana Paula PERCICOTE, Thaísa NAKADOMARI, Rogério ROBES, Ricardo Rasmussen PETTERLE, Lúcia De NORONHA, Jose Luiz De GODOY

**Affiliations:** 1 Health Sciences Sector; 2 Department of Surgery, Health Sciences Sector; 3 Pathology Department, Health Sciences Sector; 4 Veterinary Medicine, Agricultural Sciences Sector; 5 Statistics, Health Sciences Sector; Federal University of Paraná, Curitiba, PR, Brazil

**Keywords:** Hepatectomy, Hematocrit, Liver, Bloodless medical and surgical procedures, Hepatectomia, Hematócrito, Período pós-operatório, Preservação de sangue

## Abstract

**Background:**

Hepatectomies promote considerable amount of blood loss and the need to administrate blood products, which are directly linked to higher morbimortality rates. The blood-conserving hepatectomy (BCH) is a modification of the selective vascular occlusion technique. It could be a surgical maneuver in order to avoid or to reduce the blood products utilization in the perioperative period.

**Aim:**

To evaluate in rats the BCH effects on the hematocrit (HT) variation, hemoglobin serum concentration (HB), and on liver regeneration.

**Methods:**

Twelve Wistar rats were divided into two groups: control (n=6) and intervention (n=6). The ones in the control group had their livers partially removed according to the Higgins and Anderson technique, while the rats in the treatment group were submitted to BCH technique. HT and HB levels were measured at day D0, D1 and D7. The rate between the liver and rat weights was calculated in D0 and D7. Liver regeneration was quantitatively and qualitatively evaluated.

**Results:**

The HT and HB levels were lower in the control group as of D1 onwards, reaching an 18% gap at D7 (p=0.01 and p=0.008, respectively); BCH resulted in the preservation of HT and HB levels to the intervention group rats. BCH did not alter liver regeneration in rats.

**Conclusion:**

The BCH led to beneficial effects over the postoperative HT and serum HB levels with no setbacks to liver regeneration. These data are the necessary proof of evidence for translational research into the surgical practice.

**Abstract:**

A) Unresected liver; B) liver appearance after the partial hepatectomy (1=vena cava; 2=portal vein; 3=hepatic vein; 4=biliary drainage; 5=hepatic artery)

## RESUMO

**Racional:**

As hepatectomias compreendem considerável perda sanguínea e utilização de hemoderivados, o que diretamente estão relacionados com maior morbimortalidade. A hepatectomia hemoconservadora (HH) é modificação da técnica de oclusão vascular seletiva em hepatectomia. Ela pode ser alternativa cirúrgica para evitar ou diminuir o uso de hemoderivados no perioperatório.

**Objetivo:**

Avaliar os efeitos da HH sobre o volume globular (VG), concentração de hemoglobina (HB) e sobre a regeneração hepática em ratos.

**Métodos:**

Dois grupos de ratos Wistar foram constituídos: controle (n=6) e intervenção (n=6). Os do grupo controle foram submetidos à hepatectomia parcial de Higgins e Anderson e os do grupo Intervenção à HH. VG e HB foram medidos nos dias D0, D1 e D7. A relação peso do fígado/peso do rato foi calculada em D0 e D7. A regeneração hepática foi analisada qualitativamente e quantitativamente.

**Resultados:**

Houve diminuição dos níveis de VG e HB nos ratos do grupo controle a partir de D1, atingindo decréscimo de 18% em D7 (p=0,01 e p=0,008 respectivamente); a HH permitiu a manutenção dos níveis de VG e HB nos ratos do grupo intervenção. A HH não alterou a regeneração hepática.

**Conclusão:**

HH resultou em níveis maiores de VG e HB pós-operatórios sem alterar a regeneração hepática. Pode-se considerar estes dados como a prova necessária para a translação à pesquisa clinicocirúrgica.

## INTRODUCTION

The liver is a particularly complex organ. An actual chemical laboratory, it is responsible for more than 5000 functions in the body^6^. It is the sole organ that is supplied by two distinct blood supplies, being that: splanchnic, out of the portal vein, and systemic, out of the coeliac trunk. The blood inflow from these two vessels account for 1350 ml/min, in other words, 27% of the cardiac outflow at rest, which are displayed in a pressure gradient of only 9 mmHg. The liver is a highly vascularized organ and, as a great blood reservoir, may normally contain 450 ml of blood (10% of the circulating volume) or up to 1000 ml of blood, in cases of increase in right atrium pressure. 

Bleeding in the main liver-related surgical procedures, like partial hepatectomies^11^ and liver transplantation, occurs almost inevitably, and it still represents a frightening issue if the bleeding becomes massive^9^. The rational use of blood products and the management of intraoperative hemorrhage are major concerns in the surgical practice of liver operations. This fact relies on researches that found strict correlation between higher morbimortality rates and the amount of blood loss and blood units transfused^9^ ^,^ ^12^ ^,^ ^19^. Therefore, the surgical team may resort to many techniques in order to control the patient’s blood volume, such as intraoperative cell salvage, acute normovolemic hemodilution, and vascular occlusion operations^15^.

In general, bleeding prediction depends on liver disease severity, preoperative coagulation tests, recipient’s clinical picture, donated liver histological status, and transplantation team expertise. Blood loss is frequently difficult to be measured during the liver procedures, and often it is indirectly quantified by the calculation of the blood amount needed to withhold or to reach up to a predetermined level of hematocrit or serum hemoglobin concentration^9^.

Afterwards a partial hepatectomy or liver transplantation (split-liver graft or living donor graft), the hepatic regeneration takes place. This extremely complex phenomenon reestablishes the liver weight/body weight rate. The classic model of liver regeneration research was described by Higgins and Anderson in 1931, whom performed a 2/3 partial hepatectomy on rats and observed that in seven to ten days after the procedure the liver reassumes its original weight, reestablishing the normal liver weight/body weight rate, which results in 3.58% in rats^17^.

In rats, the highest DNA synthesis rate by hepatocytes - initially a quiescent cell with lifetime span of 200-400 days - occurs 24 h after the partial hepatectomy, moment at which 35% of hepatocytes are actively producing DNA-S phase of the hepatocyte cell cycle^14^. Liver regeneration is quite peculiar, because it also ensues in ex-vivo conditions. When studied under ex-vivo conditions, in an isolated-perfused liver device, the liver regeneration induced by partial hepatectomy in rats develops in a similar fashion compared to in vivo liver regeneration concerning timeframe, quantity of hepatocytes - which are actively doubling their DNA^7^ - and their lobular distribution. 

The main objective of this research was to analyze the effects of rat blood-conserving hepatectomy on hematocrit (%) and serum hemoglobin (g/dl) levels during the postoperative period. Further, we intended to evaluate the consequences of this vascular occlusion technique on liver regeneration. 

## METHODS

This research project was approved by the University Ethics Committee according to the statement number 230751662287/2017-18.

### Animals

Twelve male Wistar rats (*Rattus norvegicus albinus*), weighing 220-355g and aging 9-11 weeks old, have been placed in the laboratory of the Post-Graduation on Surgery program, where we controlled the environment temperature (20-22ºC), ventilation and light-dark cycles (12-12 h). The rats were fed ad libitum with standard feed and were supplied with common tap water. In the preoperative period, the rats have been split in groups of five animals. Each group has been placed inside identical acrylic boxes, filled with wood shavings, meanwhile, in the postoperative period, the rats have remained separate from each other in appropriately identified single boxes. 

### Surgical procedures

The operations have been accomplished under clean conditions, not sterile ones, and were carried out with microsurgery instruments. The rats were sedated with intramuscular ketamine (30 mg/kg) and xylazine (2 mg/kg) injections. They were anesthetized with isoflurane solution (1%) plus 100% oxygen inhalation at a 0.5 l/min flow. The rats were weighed at D0, D1 and D7. In addition, the resected liver lobes were weighed after the partial hepatectomy at D0; the caudate lobe biopsy was weighed at D1; and the complete liver was weighed after the total hepatectomy at D7. 

The rats’ abdomen were shaved and were cleansed with an antiseptic alcoholic solution of polyvinylpyrrolidone (10%). Then, a median laparotomy was carried out. After the procedures, the abdominal wall was sutured on two planes with nylon 4-0 (Bioline™) thread. Before suturing the abdomen, 2 ml of saline were left in the abdominal cavity in order to hydrate the rat. With the purpose of achieving postoperative analgesia, meloxicam (0.2 mg/kg) was injected in the peritoneal cavity. Besides, yohimbine (0.1 mg/kg) was injected in the peritoneal cavity to antagonize the sedation. 

#### 2/3 partial hepatectomy

**FIGURE 1:**
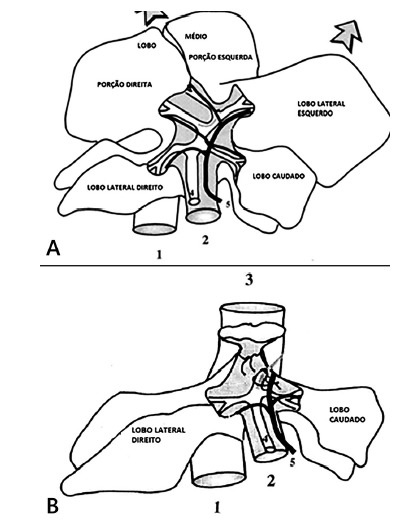
A) Unresected liver, the left lateral lobe and the median lobe were cranially displaced in order to expose the hepatic vasculature, just as the caudate and right lateral lobes; B) liver appearance after the partial hepatectomy according to Higgins and Anderson procedures: 1 - vena cava (infrahepatic part); 2 - portal vein; 3 - hepatic vein; 4 - biliary drainage; 5 - hepatic artery.

The vascular and biliary pedicles from the median lobe and left lateral lobe were isolated and ligated with a 4-0 cotton thread according to the Higgins and Anderson technique^17^ (Figure 1). The median lobe and left lateral lobe parenchyma were cut off at a level 3 mm proximal to the pedicle ligation. These two lobes were resected with all the blood contained in its interior. The parenchyma was weighed. Then, the histological structure of the resected lobes were analyzed. 

### 2/3 partial blood-conserving hepatectomy

The portal, arterial and biliary branches of the median and left lateral lobes were isolated and ligated with a 4-0 cotton surgical thread. The ligation of the lobes outflow and the harvesting of the liver parenchyma were started 15 min after the first ligation. The median lobe and left lateral lobe were harvested at a level 3 mm proximal to the pedicle ligation. The resected parenchyma was weighed and its histological structure was analyzed.

### Research design

Two groups of rats were constituted: control (n=6) and intervention (n=6). In both groups, Higgins and Anderson’s technique of partial hepatectomy was performed at D0; a liver biopsy of the caudate lobe anterior segment was sampled 24 h after the partial hepatectomy (D1); and in both groups, euthanasia procedures were carried out at D7, after the total hepatectomy. In the control group, the median and left lateral lobes were resected right after the en bloc ligation of the vascular pedicles. On the other hand, in the intervention group, the blood-conserving technique was performed during the Higgins and Anderson’s partial hepatectomy. The blood and biliary pedicles supplying the median and left lateral lobes were ligated 15 min previously to the ligation of the liver outflow and to the harvesting of the liver parenchyma. At D0, D1 and D7 blood samples were obtained by venous puncture on the infrahepatic portion of the vena cava. The samples were immediately placed in EDTA K2 (Microtainer®, BD) tubes, which were stored in a conventional freezer (4º C) for 6 h beforehand the blood analysis was led. 

#### Blood analysis

The blood parameters measurement was concluded with the aid of a Horiba™ ABX Micros 60^®^ machine after a period of normalization to the ambient temperature and homogenization of the samples for approximately 10 min in the first tube. The others have taken longer intervals in environment temperature before the analysis. Then, the hematocrit and serum hemoglobin levels were evaluated in all the samples. 

#### Qualitative histological analysis

At D0, D1 and D7 the harvested liver parenchyma samples were rapidly placed in formalin solution (10%) after weighing the material. The material was fixated, stained with H&E dye and thin histological sheaths embedded in paraffin were obtained. Each slide was examined by and independent observer with the aid of an Olympus™ 500x magnified light microscope. The modification of the liver histology was analyzed.

#### Quantitative histological analysis

Immunohistochemistry techniques were utilized to evaluate liver regeneration through observation of proliferating hepatocytes nuclei quantity. Samples were deparaffinized with 37ºC xylol solution; dehydrated in alcohol dehydrating series and rehydrated with water. Methanol and hydrogen peroxide were used as primary endogenous peroxidase blockers, and distilled water and hydrogen peroxide as secondary ones. A BioSB^®^ antigenic retrieving solution was used in 99º C water bath during 30 min. The samples were incubated overnight with 1:200 diluted mice monoclonal anti-PCNA primary antibodies (PC10 clones, DAKOT, DakoCytomation, Hostrup, Denmark). Dako Advanced Thrp System, Dako, Cytomation, Inc, CA, USA, was used for 30 min as the secondary antibody. Diaminobenzidine was used in order to reveal the chemical reactions. The slides were then counterstained with mayershematoxylin. The standard technique, which excludes the primary antibody, was employed as negative control. Positive controls were used in all reactions. Eventually, immunohistochemistry reactions were applied on 36 slides (three for each rat at D0, D1, and D7). The immunostained slides were photographed by an Axio Scan Z1 Digitizer Machine and the images were analyzed with the aid of the Image Pro Plus 4 software. 600 high power field images were taken out of each slide, approximately. From these, 30 (10 centrilobular, 10 intermediate zone, and 10 periportal zones images) were voluntarily chosen. Images with artifacts were excluded. A positive control high power field image which showed adequate levels of tissue PCNA immunoexpression was set as a mask in the software. On this image the observer manually selected brownish shades on hepatocytes nuclei - a pattern to be followed by the software. The mask then was superimposed on every other chosen image. The software was able to automatically count the quantity of nuclei stained with the anti-PCNA antibody. Average results were calculated out of all the measurements. 

### Statistical analysis

The comparison between the control and intervention groups was made according to the Mann-Whitney test. The blood analysis intragroup comparison at D0, D1 and D7 was made according to the Wilcoxon test. The results obtained from the immunohistochemistry reactions were analyzed according to variance (ANOVA) with group interaction effect (control and intervention) and zones (periportal, intermediate, and centrilobular). The data analysis was carried out with the use of the R software, version 3.4.0. The values were expressed as averages and standard deviations. P values <0.05 were considered significant.

## RESULTS

All the rats survived to the partial hepatectomy (D0) and to the caudate lobe biopsy procedure (D1). In the intervention group, the blood and biliary pedicles remained ligated for 15.3 min (±1.98) before the ligation of the liver outflow and liver resection. We excluded the results of the blood analysis of one of the rats. 

The average weighs of the rats were statistically similar in both groups at D0, D1 and D7 (Table 1). 

**TABLE 1:** Comparison of the weighs of the rats (g) at D0, D1 and D7

	Control	Intervention	p
D0	260.5 ± 22.4	291.0 ± 48.1	0.1797
D1	244.5 ± 19.1	272.9 ± 47.3	0.1797
D7	265.5 ± 22.4	289.0 ± 49.6	0.3939

Average values ± standard-deviation; p=probability of significance

The partial hepatectomy intraoperative time in the control group was 51.2 min (±8.6) while in the intervention group it resulted in 65.5 min (±11.3). We have observed more bleeding during the resection of the liver parenchyma in the control group. However, no bleeding was remarkable during the procedure performed in the intervention group rats.

**FIGURE 2:**
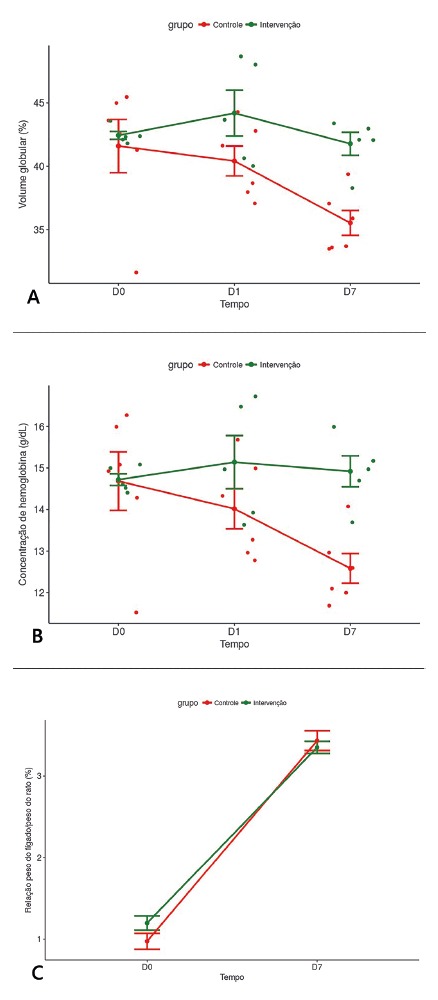
A) Distribution of hematocrit (%) levels at D0, D1 and D7; the bars represent standard-deviation that spreads from the average value; B) distribution of hemoglobin levels (g/dL) at D0, D1 and D7; the bars represent standard-deviation that spreads from the average value; C) liver/rat weights rate at D0 and D7 in control and intervention groups; at D0, residual liver weight was taken into account; at D7, the regenerated liver weight was used.

The blood-conserving hepatectomy resulted in higher hematocrit and serum hemoglobin levels (g/dl) in the postoperative period in the intervention group. This difference was already visible at D1 and so it remained until D7. In D1 nevertheless, the difference only leaned to a statistical significance, while in D7 it was clearly significant. The statistical analysis also found similarity between the groups in D0, concerning the hematocrit and serum hemoglobin levels (Figures 2A and 2B, Table 2). 

**TABLE 2:** Values of hematocrit (%) and hemoglobin levels (g/dl) at D0, D1 and D7 in control and intervention groups

	Control	Intervention	p
Ht D0	41.60 ± 5.14pp	42.44 ± 0.69pp	0.583
Ht D1	40.42 ± 2.90pp	44.20 ± 4.05pp	0.1775
Ht D7	35.53 ± 2.40pp	41.78 ± 2.03pp	0.0135
Hb D0	14.68 ± 1.72	14.72 ± 0.31	0.7144
Hb D1	14.02 ± 1.18	15.14 ± 1.43	0.2002
Hb D7	12.58 ± 0.87	14.92 ± 0.83	0.0086

Average values ± standard-deviation; Ht=hematocrit (%); Hb=hemoglobin levels (g/dl); pp=percentage points; p=probability of significance.

The time measured between the partial hepatectomy in D0 and the conclusion of the caudate lobe biopsy in D1 was 1408 min (±50.4) and 1361.8 min (±27.5) in the control and intervention groups, respectively. The rate between liver weight and rat body weight was calculated (Figure 2C). At D0, the residual liver weight/rat body weight rate was 0.97% and 1.14% in the control and intervention groups, respectively (p=0.132). At D7, the regenerated liver weight/rat body weight was 3.38% and 3.35% in the control and intervention groups, respectively (p=1.00).

**FIGURE 3:**
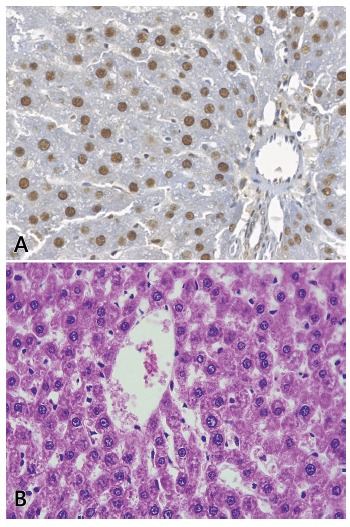
A) Central perivenular sinusoidal dilation; hepatic biopsy performed at D7 after the total hepatectomy; B) high power field image of a D1 periportal zone immunostained with anti-PCNA antibody.

Concerning the histological analysis, we found minimal alterations in the Rappaport-described liver acinars, in both groups, in all three periods (D0, D1 and D7). The most consistent outcome in all the samples was central perivenular sinusoidal dilation, which is shown in the Figure 3. Further, most of the D1 slides presented tumefaction of the central perivenular hepatocytes. 

With regards to the immunohistochemistry results, Figure 3-B depictures a high power field image of a periportal zone stained with anti-PCNA. We have not observed any statistically significant difference to the number of proliferating hepatocytes between the control and intervention groups (Table 3). Besides, we have observed that in both groups the three Rappaport-described hepatic zones have proliferated similarly.

**TABLE 3:** Proliferating hepatocytes in three different liver zones at D1 in both control and intervention groups

	Control	Intervention	p
Periportal	83.40 ± 10.11	91.97 ± 14.57	0.113
Intermediate	88.23 ± 8.92	88.93 ± 15.76	0.981
Centrilobular	82.85 ± 7.57	92.92 ± 11.90	0.588

Average values ± standard-deviation; p=probability of significance

## DISCUSSION

This research has demonstrated the advantage of blood-conserving hepatectomy on withholding the hematocrit and serum hemoglobin levels in the postoperative period of rats’ partial hepatectomies. In second place, the blood-conserving technique does not interfere with the liver regeneration and it comprises a simple procedure by which the surgeon awaits for the liver blood to be self-drained after the ligation of the main afferent branches to the resecting liver lobe.

The blood-conserving hepatectomy avoided the decrement on blood parameters (it has guaranteed hematocrit and serum hemoglobin levels 18% higher) in the postoperative period. This advantage could be crucial to keep these parameters levels above the transfusion threshold. In general, according to the American Association of Blood Banks, the hemotransfusion is indicated when the serum hemoglobin level of the stable and hospitalized patient becomes lower than 7 g/dl or 8 g/dl in orthopedics, cardiac procedures and as well as in patients with cardiovascular comorbidity^3^. Recently, however, Sean *et al* ^*1*^ - based on current discussion panels with specialists in transfusion of hepatectomized patients - published the Ottawa criteria, which states the following thresholds: 7.5 g/dl in the intraoperative period; 7 g/dl in patients without coronary artery disease in the postoperative period and 8 g/dl in patients with coronary disease in the same period; 7.5 g/dl in patients during the immediate postoperative period or a 1.5 g/dl fall on the blood panel during the postoperative period; remarkable bleeding and ST segment deviations always require transfusion.

In this research, the liver regeneration in the intervention group, evaluated by morphometric data, developed in a similar fashion in relation to the control group (Figure 2C). At D0, the weights of the residual liver lobes (right lateral and caudate lobes) were estimated out of a calculation that was made. We subtracted the weight of the resected liver/body weight rate from the reference rate of total liver weight/body weight in rats, which equals 3.58% according to a classic reference^17^. At D0, the control rats tended to result in lower residual liver weight/body weight rates compared to the invention rats. Possibly, this outcome comes from the fact that in the control group the median lobe and the left lateral lobe were fulfilled with blood at the moment they were resected, which increased the weight of the resected lobes on the scale evaluation. In the posterior 3.58% subtraction, the impression of smaller residual lobes became apparent. 

The blood-conserving hepatectomy is a kind of vascular occlusion procedure in liver surgery. The diverse sorts of liver vascular occlusion, being total or selective, envisage mainly reducing the blood loss during the procedure. Current systematic reviews by Hoekstra *et al* ^*18*^ points out the ideal patient to each one of the different techniques. According to them: if the predicted blood loss is not important, no vascular occlusion should be endeavored; on the contrary, continuous Pringle maneuver should be used in healthy organs and intermittent Pringle maneuver on cirrhotic parenchyma; tumors resection that involve the vena cava must be made resorting to total hepatic vascular exclusion, if possible with hypothermic solutions in order to reduce liver and renal function hazards during the postoperative period; in patients that do not support the hemodynamic dysfunction induced by the total hepatic vascular exclusion or patients in which the Pringle maneuver was not sufficient to contain the hemorrhage, selective hepatic vascular occlusion might be used. Nevertheless, in none of the vascular occlusion papers researched^2^ ^,^ ^4^ ^,^ ^8^ ^,^ ^10^ ^,^ ^15^ there is the concept of retrieving the blood imprisoned in the liver parenchyma that would be resected. In none of these papers, the hematocrit or serum hemoglobin levels were measured during the postoperative period. 

The blood loss amount and blood products utilization in the perioperative period are linked to higher morbimortality rates^19^. Because of this ascertainment, a myriad of authors has already published papers whose main topics were surgical interventions (like vascular occlusion or harmonic scalpel) and anesthetic protocols that prevent or reduce the necessity to use blood transfusion in hepatectomies. Sima *et al* ^*23*^ developed an algorithm that predicts with 70% of accuracy the need to transfuse a patient during the perioperative period of a liver surgery. Recent paper^20^ utilized this and other two transfusion prediction tools^5^ ^,^ ^21^ to elaborate another simplified tool which comprises three criteria: anemia, extension of the liver resection and primary malignancy of the liver disease. These instruments allow the surgeon to better choose the most appropriate technique and allow the anesthesiologist to enroll the patient to the best protocol (autologous preoperative blood donation, acute normovolemic hemodilution, intraoperative cell salvage) in order to save blood bank resources.

When the surgical team is not able to conserve the patient’s hematocrit, inevitably one has to ponder about transfusing blood products. Although the better donor selection, like triage of possible infected donors, has diminished the probability of a hazardous outcome, potential risks still do exist. According to Goodnough^13^, the most frequent adverse reactions related to the transfusion practice are: excess of fluid infusion (1 in 20 units) that might cause acute pulmonary edema if the transfusion proceeds too rapidly; mild allergic reactions (1 in 30-100 units) and non-hemolytic febrile reaction (1 in 20-200 units). One must also reason there may exist a paucity of resources in the blood bank, besides the acknowledgement of blood products costs to the health system. A 2015 Australian research stated that transfused patients cost 1.83x more than non-transfused patients^24^. Another research conducted in the USA in 2009 points out an average cost (considering all steps from the harvesting to the infusion indeed) of USD 761,00 (+/-294,00) per unit of packed red cells^22^.

However, one cannot precise if the technique was able to rescue this volume of blood completely. An appreciable perspective with the aim to optimize the technique - and retrieve the total imprisoned blood - is the possibility to rinse the lobe to be resected with saline before the distal ligation of the blood and biliary pedicles. After the selective ligation of the portal and arterial branch to the lobes to be resected, one could consider to catheterize these vessels and perfuse the lobe with saline until the liver parenchyma shows itself emptied of blood. This perspective is difficult to be performed in an animal as small as a rat. Nevertheless, the data obtained in this research allows one to design a new surgical protocol to be conducted in centers that accomplish large volumes of liver transplantation procedures as well as hepatectomies. 

## CONCLUSION

The blood-conserving hepatectomy allowed the maintenance of hematocrit and serum hemoglobin levels at the expense of retrieving the blood imprisoned in the resected lobe.
